# Weight Recurrence After Bariatric Surgery: Incretin-Based Therapies and the Evolving Role of Revisional Surgery

**DOI:** 10.7759/cureus.113460

**Published:** 2026-07-27

**Authors:** Luis A Huerta Díaz, Brandon A Lopez Alanis, Luis F Zorrilla-Nuñez, Gerardo E Muñoz-Maldonado

**Affiliations:** 1 General Surgery, Hospital Universitario “Dr. José E. González” UANL, Monterrey, MEX

**Keywords:** glp-1 receptor agonists, incretin-based therapy, metabolic and bariatric surgery (mbs), obesity pharmacotherapy, revisional bariatric surgery, weight recurrence

## Abstract

Weight recurrence following metabolic and bariatric surgery (MBS) represents a clinically significant challenge that reflects the chronic and relapsing nature of obesity. Although revisional bariatric surgery has traditionally been considered the primary treatment option for affected patients, it is associated with greater technical complexity and higher complication rates than primary procedures. The emergence of highly effective incretin-based pharmacotherapies, including glucagon-like peptide-1 receptor agonists (GLP-1 RAs) and the dual glucose-dependent insulinotropic polypeptide (GIP)/GLP-1 RA tirzepatide, has substantially broadened the treatment options available for this population.

This review examines the epidemiology, pathophysiology, and clinical evaluation of post-operative weight recurrence and explores the evolving role of incretin-based therapies as potential alternatives or adjuncts to revisional bariatric surgery. Current evidence indicates that post-operative weight recurrence is multifactorial, arising from neurohormonal adaptation, behavioral factors, metabolic compensation, and anatomical changes. Randomized trials, systematic reviews, meta-analyses, and observational studies consistently demonstrate that GLP-1 RAs can achieve clinically meaningful weight loss in patients with insufficient weight loss or post-operative weight recurrence, particularly in the absence of correctable anatomical abnormalities. Tirzepatide has demonstrated greater weight-loss efficacy than earlier incretin-based therapies and may represent an important therapeutic option for appropriately selected post-bariatric patients. Emerging agents, including triple-RAs, GLP-1/glucagon dual agonists, and oral GLP-1 RAs, may further expand future treatment options.

Collectively, the available evidence supports a multidisciplinary, step-wise management strategy incorporating anatomical assessment, lifestyle optimization, and evidence-based pharmacotherapy before consideration of revisional surgery. While revisional procedures remain essential for patients with anatomical failure, modern incretin-based therapies may reduce, delay, or better select the need for revisional surgery in appropriately evaluated patients. Further prospective studies are needed to define their long-term role within post-bariatric treatment algorithms.

## Introduction and background

Obesity has emerged as one of the most pressing global health challenges of the twenty-first century, affecting over 1 billion individuals worldwide and contributing substantially to morbidity, mortality, and healthcare expenditures. The condition is associated with numerous obesity-related disorders, including type 2 diabetes mellitus, hypertension, dyslipidemia, obstructive sleep apnea, cardiovascular disease, metabolic dysfunction-associated steatotic liver disease, and several malignancies [1,2]. Despite advances in lifestyle interventions and pharmacotherapy, long-term weight reduction remains difficult to sustain in patients with severe obesity. Consequently, MBS has become the most effective and durable treatment for obesity and its associated conditions, producing substantial and sustained weight loss, improvement of metabolic disorders, and reductions in obesity-related mortality [1].

Utilization of bariatric surgery has increased substantially over the past two decades, with sleeve gastrectomy (SG) and Roux-en-Y gastric bypass (RYGB) representing the most frequently performed procedures worldwide [1]. Numerous studies have demonstrated superior long-term weight-loss outcomes following bariatric surgery compared with non-surgical interventions [1,3]. Nevertheless, long-term success is not universal. A substantial proportion of patients experience either insufficient weight loss or post-operative weight recurrence, which may erode the metabolic benefits achieved after surgery and adversely affect quality of life. Although suboptimal adherence to recommended dietary and lifestyle modifications may contribute in some patients, post-operative weight recurrence is now recognized as a multifactorial process involving behavioral, neurohormonal, metabolic, psychological, and anatomical factors [4-7].

Establishing the true prevalence of post-operative weight recurrence remains challenging because universally accepted definitions are lacking [4-6]. Reported rates vary substantially across studies according to the criteria applied and the duration of follow-up, limiting direct comparisons and interpretation of outcomes [4,5]. Long-term observational studies consistently demonstrate that recurrence becomes increasingly common beyond five years after surgery, underscoring the chronic and relapsing nature of obesity despite successful surgical treatment [4,5].

The mechanisms underlying post-operative recurrence are multifactorial and involve complex interactions among behavioral, psychological, hormonal, metabolic, and anatomical factors [5-7]. Dietary nonadherence, reduced physical activity, maladaptive eating behaviors, psychiatric disorders, metabolic adaptation, gastric pouch enlargement, sleeve dilation, gastro-gastric fistula formation, and gastrojejunal anastomotic dilation have all been implicated [5,6]. Consequently, evaluation of patients presenting with recurrence requires a comprehensive multidisciplinary approach integrating nutritional, psychological, endoscopic, radiologic, and surgical assessment [2,4].

Historically, revisional bariatric surgery represented the principal therapeutic option for patients with clinically significant post-operative recurrence, particularly when anatomical abnormalities were identified [4,8]. Although revisional procedures may provide meaningful additional weight loss, they are technically more demanding than primary operations and are associated with increased operative times, higher complication rates, longer hospital stays, and greater healthcare costs [4,8]. These limitations have prompted growing interest in less invasive strategies capable of restoring weight loss while minimizing procedural risk.

The introduction of incretin-based pharmacotherapies has transformed the management of obesity and its recurrence after bariatric surgery. GLP-1 receptor agonists (GLP-1 RAs), including liraglutide and semaglutide, have demonstrated substantial efficacy in promoting weight loss through appetite suppression, delayed gastric emptying, enhanced satiety, and favorable metabolic effects [9]. More recently, the dual glucose-dependent insulinotropic polypeptide (GIP)/GLP-1 RA tirzepatide has produced even greater weight reduction than previously available pharmacologic therapies [10,11]. Emerging evidence suggests that these agents may provide an effective treatment strategy for patients experiencing insufficient weight loss or post-operative recurrence following bariatric surgery [12-17].

As increasingly effective obesity management medications (OMMs) become available, the traditional paradigm in which revisional surgery was considered the inevitable next step after post-operative recurrence is being reconsidered [4,8]. Current evidence increasingly supports a step-wise management strategy incorporating multidisciplinary evaluation, optimization of lifestyle and behavioral interventions, identification of anatomical abnormalities, and evidence-based pharmacotherapy before revisional procedures are pursued [4,8,11]. Nevertheless, important questions remain regarding patient selection, treatment timing, expected outcomes, and the extent to which incretin-based therapies may ultimately reduce the need for reoperation.

Literature search strategy and scope of the review

A literature search was conducted using PubMed, Scopus, and Embase through June 2026. Search terms included combinations of "bariatric surgery," "metabolic surgery," "weight recurrence," "weight regain," "GLP-1 receptor agonists," "semaglutide," "tirzepatide," "obesity pharmacotherapy," and "revisional bariatric surgery." Priority was given to randomized controlled trials, systematic reviews, meta-analyses, clinical practice guidelines, position statements, and high-quality observational studies. The literature search strategy is summarized in Table 1.

**Table 1:** Literature search strategy Summary of the databases searched, search terms used, and study types prioritized for inclusion in this narrative review MBS: Metabolic and bariatric surgery

Component	Details
Databases searched	PubMed, Scopus, Embase
Search period	Through June 2026
Search terms	"bariatric surgery," "metabolic surgery," "weight recurrence," "weight regain," "GLP-1 receptor agonists," "semaglutide," "tirzepatide," "obesity pharmacotherapy," "revisional bariatric surgery"
Study types prioritized	Randomized controlled trials, systematic reviews, meta-analyses, clinical practice guidelines, position statements, high-quality observational studies
Inclusion focus	Epidemiology, pathophysiology, clinical evaluation, and incretin-based pharmacotherapy in post-operative weight recurrence after MBS

This review examines the epidemiology and pathophysiology of post-operative weight recurrence, the contemporary framework for patient evaluation, and the emerging evidence supporting incretin-based therapies following bariatric surgery. Particular emphasis is placed on the potential role of GLP-1 RAs and dual incretin agonists as therapeutic alternatives or adjuncts to revisional bariatric surgery. The objective is to evaluate whether modern obesity pharmacotherapy may alter the traditional treatment algorithm and reduce, delay, or better stratify the need for revisional intervention in appropriately selected patients. 

## Review

Pathophysiology of weight recurrence after bariatric surgery

Weight recurrence after MBS is a complex phenomenon that cannot be explained solely by technical failure or patient non-adherence. Contemporary evidence increasingly supports the concept that obesity is a chronic, relapsing disease governed by biological mechanisms that actively defend body weight and promote restoration following weight loss [4,9,10]. Although bariatric surgery induces profound anatomical, hormonal, and metabolic changes that facilitate substantial weight reduction, many physiological pathways involved in energy homeostasis remain active and may contribute to long-term recurrence. Importantly, the complex metabolic adaptations underlying both durable weight loss and post-operative weight recurrence are not yet fully understood, and continue to be an area of active investigation [9,10].

Neurohormonal Adaptation and Energy Homeostasis

Body weight regulation involves an intricate network encompassing the hypothalamus, brainstem, gastrointestinal tract, adipose tissue, pancreas, and peripheral nervous system [9]. Weight loss - whether achieved through lifestyle modification, pharmacotherapy, or surgery - triggers compensatory mechanisms designed to restore energy balance, including increased hunger, reduced satiety, alterations in reward signaling, and decreased energy expenditure [9,10].

Following bariatric surgery, patients typically experience favorable changes in gut hormones involved in appetite regulation, including glucagon-like peptide-1 (GLP-1), peptide YY (PYY), oxyntomodulin, and cholecystokinin [9]. These hormonal adaptations contribute significantly to early post-operative success by enhancing satiety and reducing caloric intake. Long-term studies suggest, however, that variability in hormonal responses may partially explain divergent weight trajectories among patients over time [10].

The Role of GLP-1 in Post-operative Weight Regulation

GLP-1 is an incretin hormone secreted by enteroendocrine L-cells located primarily in the distal small intestine and colon. Beyond its established effects on glucose homeostasis, GLP-1 exerts potent anorexigenic actions through both central and peripheral mechanisms [9]. Activation of GLP-1 receptors within hypothalamic appetite-regulating centers promotes satiety, reduces hunger, delays gastric emptying, and decreases food intake [17-19].

One of the most clinically significant physiological effects of RYGB and SG is the enhancement of endogenous GLP-1 secretion following nutrient ingestion [9,10]. Increased post-prandial GLP-1 concentrations are believed to contribute substantially to post-operative weight loss and metabolic improvement. Emerging evidence suggests that patients who experience post-operative recurrence may exhibit attenuated GLP-1 responses compared with those who maintain durable weight loss, supporting the hypothesis that impaired incretin signaling contributes to long-term recurrence [10].

These observations help explain why GLP-1 RAs may be effective in patients with post-operative recurrence. By pharmacologically augmenting pathways that are naturally enhanced after bariatric surgery, agents such as liraglutide and semaglutide may help restore satiety signaling and counteract adaptive mechanisms that favor weight restoration [11-15].

Behavioral and Psychological Factors

Although biological mechanisms play a central role, behavioral and psychological factors remain important contributors to post-operative recurrence [5,6]. Maladaptive eating behaviors - including grazing, binge eating, emotional eating, and loss-of-control eating - have been consistently associated with poorer long-term outcomes [5]. Inadequate physical activity, poor dietary adherence, substance use disorders, and limited post-operative follow-up further increase the risk of recurrence [5,6].

Psychiatric disorders, including depression, anxiety disorders, and untreated eating disorders, have also been associated with less favorable outcomes [5]. These factors frequently coexist with biological drivers of recurrence, reinforcing the need for multidisciplinary assessment and individualized treatment planning.

Metabolic Adaptation

Adaptive thermogenesis represents another important contributor to long-term recurrence. Following substantial weight loss, total energy expenditure decreases beyond what would be predicted on the basis of changes in body composition alone [9]. This metabolic adaptation creates a persistent environment favoring weight restoration by increasing energy efficiency and reducing caloric requirements. Similar responses have been documented after both surgical and non-surgical weight-loss interventions and may persist for years [9], further reinforcing the concept of obesity as a chronic disease requiring lifelong management.

Anatomical Factors

Anatomical abnormalities may also contribute to post-operative recurrence and should be considered during patient evaluation [4]. In patients who have undergone RYGB, gastric pouch dilation, enlargement of the gastrojejunal anastomosis, and gastro-gastric fistula formation have all been associated with recurrent weight gain [20,21]. Among patients who have undergone SG, progressive sleeve dilation, residual fundus enlargement, or technical factors that reduce long-term restriction may contribute similarly [21].

Nevertheless, anatomical abnormalities account for only a subset of patients presenting with post-operative recurrence. Many individuals demonstrate no identifiable structural abnormalities despite substantial recurrent weight gain, highlighting the predominant role of neurohormonal, metabolic, and behavioral mechanisms in long-term weight regulation [4,5].

A Multifactorial Model

**Figure 1:**
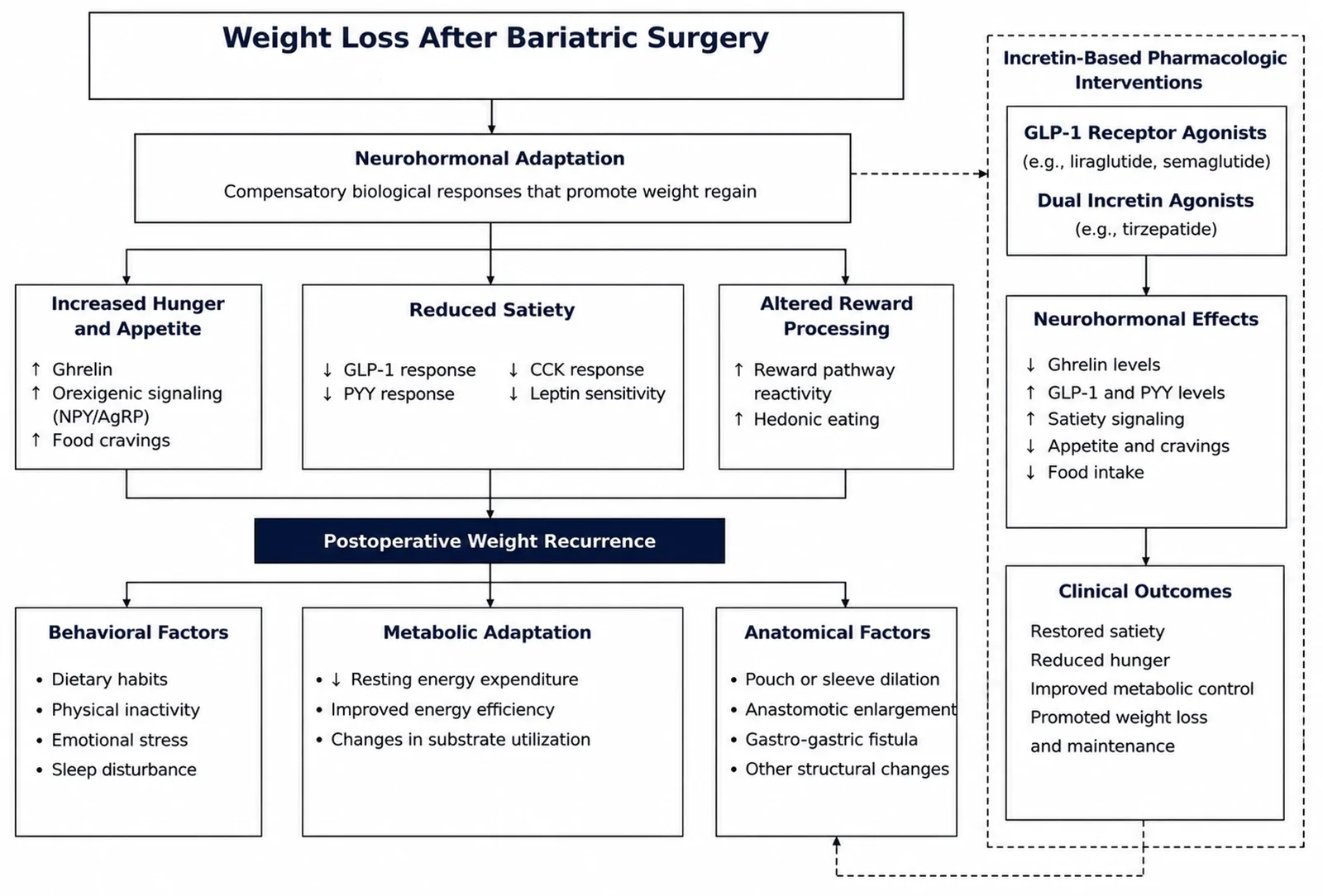
Multifactorial model of post-operative weight recurrence and the therapeutic role of incretin-based pharmacotherapy Post-operative weight recurrence results from the interaction of neurohormonal adaptation, behavioral factors, metabolic compensation, and anatomical changes following bariatric surgery [4,6,9]. Attenuated satiety signaling, increased appetite, altered reward processing, and adaptive reductions in energy expenditure contribute to restoration of body weight [9,10]. GLP-1 RAs and dual incretin agonists counteract several of these mechanisms by enhancing satiety, reducing hunger, improving neurohormonal signaling, and promoting sustained weight loss and metabolic control [11,18]. Original figure created by the authors based on concepts described in [4,9,10]. GLP-1 RA: Glucagon-like peptide-1 receptor agonist

Current evidence supports a multifactorial model in which biological, behavioral, metabolic, and anatomical factors interact to influence long-term outcomes after bariatric surgery [4-6]. The principal mechanisms contributing to post-operative weight recurrence and the therapeutic targets of incretin-based therapies are summarized in Figure 1.

This conceptual framework has important therapeutic implications. Patients with significant anatomical abnormalities may benefit from endoscopic or revisional surgical interventions, whereas those without correctable structural defects may be well suited for pharmacologic therapies targeting appetite regulation and energy homeostasis [4,11]. The emergence of incretin-based therapies has renewed interest in the pharmacologic management of post-operative recurrence because these agents directly target key biological pathways involved in appetite regulation and energy homeostasis.

Evaluation of patients with weight recurrence after bariatric surgery

Evaluation of patients presenting with weight recurrence after bariatric surgery requires a systematic, multidisciplinary approach. Because recurrence is frequently multi-factorial, effective management depends on identifying the relative contributions of behavioral, psychological, metabolic, hormonal, and anatomical factors rather than assuming a single underlying cause [4-6]. A thorough assessment is essential to determine whether patients are most likely to benefit from lifestyle optimization, pharmacotherapy, endoscopic intervention, or revisional surgery.

Clinical Assessment

The initial evaluation should include a detailed history addressing the timing, magnitude, and pattern of weight recurrence [4,5]. Patients should be questioned about dietary habits, physical activity, medication adherence, alcohol consumption, sleep quality, and psychosocial factors that may influence long-term weight regulation [5,6]. Particular attention should be paid to maladaptive eating behaviors - including grazing, binge eating, emotional eating, and loss-of-control eating - which are consistently associated with poorer post-operative outcomes [5].

Documentation of the patient's weight trajectory is equally important. Recording preoperative weight, nadir post-operative weight, percentage of excess weight lost, and the timing and pattern of recurrence provides valuable mechanistic insights and guides subsequent evaluation [4,18].

Nutritional Assessment

Nutritional evaluation should be performed by clinicians experienced in bariatric patient management. Dietary assessment should identify excessive caloric intake, consumption of energy-dense foods, inadequate protein intake, loss of lean body mass, frequent snacking, and other eating patterns that may contribute to recurrence [4,5].

Laboratory testing should screen for nutritional deficiencies commonly encountered after bariatric surgery, including deficiencies of iron, vitamin B12, folate, vitamin D, calcium, and thiamine [2]. Correction of these deficiencies is essential, as they may adversely affect physical capacity, quality of life, and adherence to long-term treatment.

Psychological and Behavioral Evaluation

Psychological assessment is a critical component of post-operative evaluation. Psychiatric disorders, including depression, anxiety disorders, eating disorders, and substance misuse, may substantially contribute to recurrence and should be systematically identified [5,6]. Behavioral assessment should examine eating patterns, coping strategies, emotional triggers, and adherence to recommended lifestyle modifications [5]. Patients with significant psychosocial stressors or untreated psychiatric conditions may require targeted behavioral interventions before pharmacologic or procedural therapies can achieve optimal results.

Assessment of Metabolic and Endocrine Factors

Secondary metabolic and endocrine causes should be considered during evaluation, even though obesity itself is characterized by biological adaptations that favor restoration of body weight [4]. Thyroid dysfunction, Cushing syndrome, and medication-induced weight gain may contribute to post-operative recurrence and should be investigated when clinically indicated [3]. Assessment of obesity-related disorders - including type 2 diabetes, hypertension, dyslipidemia, and obstructive sleep apnea - is equally important, as their recurrence may accompany weight recurrence and influence treatment selection [4].

Anatomical Evaluation

Anatomical assessment is particularly relevant in patients with substantial recurrence, failure of medical therapy, or symptoms suggesting structural abnormalities [4,21]. Upper gastrointestinal contrast studies, upper endoscopy, CT, and other imaging modalities may be used to characterize post-operative anatomy and identify potentially correctable causes of recurrence [21].

Common findings in patients who have undergone RYGB include gastric pouch enlargement, dilation of the gastrojejunal anastomosis, and gastro-gastric fistula formation [20,21]. Following SG, sleeve dilation, residual fundus enlargement, and technical variations in sleeve construction may be identified [21]. A significant proportion of patients presenting with post-operative recurrence, however, demonstrate no identifiable anatomical abnormalities despite thorough evaluation [4,21]. This observation reinforces the central role of neurohormonal and behavioral mechanisms and highlights the potential value of pharmacotherapy in appropriately selected patients.

Determining the Optimal Therapeutic Strategy

The ultimate goal of evaluation is to identify the dominant contributors to recurrence and select the most appropriate intervention. Patients with significant anatomical abnormalities may benefit from endoscopic revision or revisional surgery, whereas those without correctable structural defects are often more appropriately managed with intensive lifestyle intervention and OMMs [4,20,21].

The availability of highly effective incretin-based therapies has substantially expanded the therapeutic options available for patients with post-operative recurrence. Historically, revisional surgery was often considered the primary treatment option for patients with substantial recurrence. Accumulating evidence now suggests that many patients without major anatomical abnormalities may achieve significant additional weight loss through pharmacotherapy, potentially avoiding or delaying reoperation [11-15].

A Multidisciplinary Framework for Decision-Making

Current guidelines and expert consensus statements emphasize that management of post-operative recurrence should be individualized and multidisciplinary [2,4]. Bariatric surgeons, obesity medicine specialists, endocrinologists, dietitians, psychologists, and gastroenterologists may all contribute meaningfully to evaluation and treatment planning.

Rather than viewing revisional surgery and pharmacotherapy as competing strategies, contemporary management increasingly recognizes them as complementary components of a stepwise treatment algorithm, in which identification of anatomical abnormalities, optimization of lifestyle factors, and utilization of modern OMMs are integrated to maximize long-term outcomes while minimizing unnecessary procedural risk [4].

GLP-1 RAs for weight recurrence after bariatric surgery

Recognition that post-operative weight recurrence is influenced not only by behavioral and anatomical factors but also by persistent neurohormonal adaptations has increased interest in pharmacologic therapies targeting appetite regulation and energy homeostasis [9,10]. Among currently available OMMs, GLP-1 RAs have emerged as the most extensively studied pharmacologic option for patients experiencing insufficient weight loss or post-operative weight recurrence after bariatric surgery [8,11].

GLP-1 RAs act through multiple complementary mechanisms, including enhancement of satiety, reduction of hunger, delayed gastric emptying, and modulation of central reward pathways involved in food intake [9,17]. These mechanisms are particularly relevant in post-bariatric patients because they reinforce physiological pathways that are naturally augmented after RYGB and SG [9,10]. Pharmacologic activation of GLP-1 signaling therefore represents a biologically plausible strategy for restoring weight control in patients who experience recurrence despite initially successful surgery.

Liraglutide

Liraglutide was the first GLP-1 RA approved specifically for chronic weight management and has been the most extensively studied OMM in post-bariatric populations [8,11]. Early observational studies demonstrated that liraglutide could produce significant additional weight loss in patients with insufficient weight loss or post-operative weight recurrence [8].

Subsequent randomized evidence strengthened this foundation. The BARI-OPTIMISE trial demonstrated that liraglutide produced significantly greater weight reduction compared with placebo in patients with suboptimal outcomes following bariatric surgery [15]. Similarly, a randomized study evaluating liraglutide in patients with persistent or recurrent type 2 diabetes after bariatric surgery reported favorable effects on both weight control and glycemic outcomes, further supporting the therapeutic role of GLP-1 receptor agonism in this context [16].

Although liraglutide represented an important advance in the pharmacologic management of post-operative recurrence, its efficacy is generally considered inferior to that of newer agents within the same class, particularly semaglutide [13].

Semaglutide

Semaglutide represents a significant advance in obesity pharmacotherapy, offering greater weight-loss efficacy than earlier GLP-1 RAs [17]. In the Semaglutide Treatment Effect in People with Obesity (STEP-1 trial), semaglutide established a new benchmark for medical obesity treatment, demonstrating substantial weight reduction in adults with obesity [17]. Although this landmark trial excluded post-bariatric patients, its results generated considerable interest in applying semaglutide to individuals experiencing post-operative weight recurrence.

Subsequent observational studies have consistently reported favorable outcomes with semaglutide in post-bariatric populations [11-14]. Comparative analyses suggest that semaglutide achieves greater weight reduction than liraglutide among patients with post-operative recurrence, further supporting its growing role as a preferred pharmacologic option in clinical practice [13].

Evidence From Systematic Reviews and Meta-Analyses

The accumulating evidence supporting GLP-1 RAs in post-bariatric populations has been synthesized in several systematic reviews and meta-analyses [11,12]. Key studies evaluating incretin-based therapies in this population are summarized in Table 2.

**Table 2:** Clinical evidence supporting incretin-based therapies for post-bariatric weight recurrence Summary of key studies evaluating GLP-1 RAs, dual incretin agonists, and other OMMs in patients with insufficient weight loss or post-operative weight recurrence following MBS. Numbers in brackets correspond to references cited in the References section. GLP-1 RA: Glucagon-like peptide-1 receptor agonist; MBS: Metabolic and bariatric surgery; OMM: Obesity management medication; SG: Sleeve gastrectomy

Study	Year	Study Design	Population	Intervention	Main Findings
Miras et al. [16]	2019	Randomized, double-blind, placebo-controlled trial	Patients with persistent or recurrent type 2 diabetes after bariatric surgery	Liraglutide vs placebo	Liraglutide improved glycemic control and promoted additional weight reduction compared with placebo.
Jensen et al. [12]	2023	Retrospective observational study	Patients with post-operative weight recurrence	Liraglutide or semaglutide	Both agents resulted in clinically meaningful weight loss and recovery of a substantial proportion of regained weight.
Murvelashvili et al. [13]	2023	Retrospective comparative cohort	Patients with post-operative weight recurrence	Semaglutide vs liraglutide	Semaglutide achieved greater weight reduction than liraglutide.
Mok et al. (BARI-OPTIMISE) [15]	2023	Randomized controlled trial	Patients with suboptimal weight loss after bariatric surgery	Liraglutide vs placebo	Liraglutide was superior to placebo for additional weight reduction after bariatric surgery.
Dutta et al. [11]	2024	Systematic review and meta-analysis	Multiple post-bariatric populations	GLP-1 RAs	GLP-1 RAs were associated with significant additional weight loss and favorable metabolic outcomes.
Jamal et al. [14]	2024	Retrospective cohort study	Patients with weight recurrence after SG	Semaglutide or tirzepatide	Both therapies produced clinically significant weight loss; tirzepatide demonstrated greater efficacy.
Jastreboff et al. (SURMOUNT-1) [22]	2022	Randomized controlled trial	Adults with obesity	Tirzepatide	Demonstrated unprecedented weight loss compared with previous pharmacologic therapies.
Aronne et al. (SURMOUNT-5) [23]	2025	Randomized controlled trial	Adults with obesity	Tirzepatide vs semaglutide	Tirzepatide achieved greater weight loss than semaglutide.

Collectively, these studies indicate that GLP-1 RAs are associated with significant additional weight loss in patients with insufficient weight loss or postoperative weight recurrence following bariatric surgery. Available evidence suggests that pharmacologic treatment may allow many patients to recover a substantial proportion of the weight regained after surgery [11]. Although most studies are observational and vary in design, patient selection, and outcome definitions, the overall direction of evidence has been remarkably consistent in demonstrating meaningful clinical benefit [11,12].

The safety profile of GLP-1 RAs in post-bariatric populations appears broadly comparable to that observed in non-surgical patients [11]. Gastrointestinal adverse effects remain the most commonly reported side effects and are generally manageable through gradual dose escalation and appropriate patient counseling [17].

Limitations of Current Evidence

Several limitations should be acknowledged when interpreting the current literature on pharmacologic management of post-operative weight recurrence. Much of the available evidence consists of retrospective observational studies, introducing the possibility of selection bias, confounding, and variability in treatment allocation [11,12]. Outcome definitions remain inconsistent across studies, particularly regarding insufficient weight loss, weight regain, and weight recurrence, which complicates direct comparisons and limits the ability to synthesize findings across cohorts [4,18].

In addition, most published studies include relatively small sample sizes and limited follow-up durations, restricting assessment of long-term treatment durability and safety [11,12]. Direct comparative studies evaluating pharmacotherapy against revisional surgery are largely absent, and randomized controlled trials specifically enrolling post-bariatric populations remain scarce [4]. Furthermore, existing evidence is derived predominantly from patients who underwent RYGB or SG, limiting generalizability to other bariatric procedures.

Consequently, although current evidence supports GLP-1 RAs and other incretin-based therapies as effective treatment options for post-operative weight recurrence, their precise position within long-term treatment algorithms continues to evolve. Additional prospective studies are needed to identify optimal patient selection criteria, treatment duration, sequencing with endoscopic or surgical interventions, and long-term outcomes.

Clinical Implications

Despite these limitations, available evidence supports the integration of incretin-based pharmacotherapy into the multidisciplinary management of patients experiencing insufficient weight loss or post-operative weight recurrence after bariatric surgery [8,11-15]. In patients without significant anatomical abnormalities, these agents provide a minimally invasive therapeutic option capable of producing meaningful additional weight loss while potentially reducing exposure to the risks associated with revisional procedures.

Current evidence increasingly supports a stepwise treatment approach in which anatomical evaluation, nutritional optimization, behavioral interventions, and pharmacotherapy are considered before revisional surgery in appropriately selected patients [4,8,21]. Rather than replacing revisional surgery, modern OMMs expand the therapeutic armamentarium available to clinicians and may help identify those patients most likely to benefit from procedural intervention.

The success of liraglutide and semaglutide in post-bariatric populations has also provided proof of concept for incretin-based therapy as a broader treatment strategy. Emerging agents such as tirzepatide and next-generation multi-RAs may further enhance outcomes and continue to reshape the management paradigm for post-operative weight recurrence in the coming years.

Dual incretin agonists and emerging therapies

Tirzepatide: A New Paradigm in Obesity Pharmacotherapy

Tirzepatide has marked a meaningful shift in obesity pharmacotherapy, considerably widening the options available to patients with post-operative weight recurrence [22,23]. Unlike conventional GLP-1 RAs, tirzepatide acts as a dual agonist of both GIP and GLP-1 receptors, producing complementary effects on appetite regulation, energy balance, and metabolic control [22].

The rationale for dual incretin agonism rests on the synergistic actions of these two signaling pathways. While GLP-1 receptor activation promotes satiety, delays gastric emptying, and reduces food intake, GIP receptor stimulation appears to enhance metabolic regulation and augment weight-loss responses when combined with GLP-1 agonism [22]. The resulting degree of weight reduction exceeds that previously achieved with available pharmacologic therapies and narrows the efficacy gap between medical and surgical obesity treatment [22].

Evidence From Non-Surgical Obesity Populations

The SURMOUNT-1 trial established tirzepatide as one of the most effective pharmacologic therapies currently available for obesity management [22]. Participants receiving tirzepatide achieved substantially greater weight reduction than had been reported with earlier OMMs, demonstrating that modern incretin-based therapies can achieve levels of weight loss previously considered unattainable with pharmacotherapy alone [22].

The SURMOUNT-5 trial subsequently compared tirzepatide directly with semaglutide and demonstrated superior weight-loss efficacy with tirzepatide [23]. These findings are particularly relevant when considering treatment escalation in patients who have already undergone bariatric surgery and continue to experience post-operative recurrence.

Emerging Evidence in Post-bariatric Populations

Although the evidence base for tirzepatide in post-bariatric populations remains smaller than that for semaglutide and liraglutide, early data are encouraging [14]. Observational studies suggest that tirzepatide may achieve significant additional weight loss in patients experiencing recurrence after SG, with efficacy that appears to exceed that observed with semaglutide [14].

The growing clinical interest in tirzepatide reflects an important shift in therapeutic thinking. Historically, patients with substantial post-operative recurrence and no correctable anatomical abnormality had limited options beyond lifestyle modification or reoperation. The availability of dual incretin agonists has created a new treatment category capable of addressing the neurohormonal mechanisms underlying recurrence while avoiding the risks associated with revisional surgery [4,14].

Potential Advantages Over Revisional Surgery

Revisional bariatric surgery remains an important treatment option for patients with anatomical failure, including dilated gastric pouches, enlarged gastrojejunal anastomosis, gastro-gastric fistulas, and significant sleeve dilation [19-21]. However, revisional procedures are technically more complex and are associated with higher complication rates than primary operations [4,21].

Tirzepatide, by contrast, offers a non-invasive therapeutic strategy targeting the biological drivers of obesity recurrence [22]. While pharmacotherapy cannot correct structural abnormalities, many patients presenting with post-operative recurrence demonstrate no identifiable anatomical cause during evaluation [4,21]. In such cases, dual incretin agonism represents an attractive escalation strategy before revisional surgery is considered. These approaches should be viewed as complementary rather than competing interventions, with pharmacotherapy favored when neurohormonal and behavioral mechanisms predominate and revisional surgery reserved for structural failure.

Future Directions: Next-Generation Therapies

The rapid evolution of obesity pharmacotherapy suggests that the therapeutic landscape will continue to change substantially. Several investigational agents have demonstrated promising results in clinical trials, including retatrutide, a triple agonist targeting GLP-1, GIP, and glucagon receptors; survodutide, a dual GLP-1/glucagon RA; and orforglipron, an oral non-peptide GLP-1 RA [24-26]. These therapies have demonstrated impressive weight-loss efficacy in non-surgical obesity populations and may further expand treatment options for post-bariatric patients in the future.

Although evidence in post-operative populations remains limited, these developments raise the possibility that future pharmacologic interventions may further reduce the need for revisional procedures in selected patients. Additional studies will be required to determine their effectiveness, safety, and optimal positioning within post-bariatric treatment algorithms.

Implications for the Post-bariatric Treatment Algorithm

The development of increasingly effective incretin-based therapies has challenged the traditional model in which treatment escalation often moved directly from lifestyle intervention to revisional surgery. A growing body of literature supports a stepwise approach involving comprehensive multidisciplinary evaluation, identification of anatomical abnormalities, optimization of behavioral interventions, and initiation of evidence-based OMMs before consideration of revisional procedures [4,8,11].

Within this framework, tirzepatide and future incretin-based therapies may serve as central intermediate treatment options capable of restoring weight loss while reducing exposure to surgical risk.

Can Incretin-Based Therapies Reduce the Need for Revisional Bariatric Surgery?

Revisional bariatric surgery has historically been considered the definitive treatment for patients with substantial post-operative weight recurrence, particularly when obesity-related disorders recur or conservative management fails [4,21]. The emergence of highly effective incretin-based therapies has challenged this paradigm and raised an important clinical question: Should revisional surgery remain the default next step after post-operative recurrence?

The answer increasingly appears to depend on the mechanism underlying recurrence. Contemporary evidence indicates that postoperative recurrence results from the interaction of neurohormonal adaptation, behavioral factors, metabolic compensation, and, in a subset of patients, anatomical abnormalities [4-6]. Effective treatment therefore requires identification of the dominant contributors in each individual patient rather than a uniform treatment strategy.

Revisional Surgery Remains Essential for Anatomical Failure

There is little controversy regarding the role of revisional surgery in patients with demonstrable anatomical abnormalities. Gastric pouch enlargement, gastrojejunal anastomotic dilation, gastro-gastric fistula formation, and significant sleeve dilation constitute recognized indications for revisional or endoscopic intervention [20,21].

In these patients, pharmacologic therapy alone is unlikely to correct the underlying structural defect. Although OMMs may provide adjunctive benefit, procedural correction remains necessary when anatomy is the principal driver of recurrence [4,21].

The Therapeutic Gap in Patients Without Anatomical Abnormalities

The greatest potential impact of incretin-based therapies lies in the substantial proportion of patients who present with post-operative recurrence despite normal post-operative anatomy [4,21]. Historically, these individuals had limited treatment options, as lifestyle modification frequently produced modest and unsustained results, whereas revisional surgery offered uncertain benefit in the absence of a correctable structural abnormality [21].

Accumulating evidence suggests that recurrence in this population is primarily driven by persistent biological mechanisms governing appetite regulation and energy balance rather than technical failure of the original operation [9,10]. Under these circumstances, pharmacologic therapies targeting the same neurohormonal pathways implicated in obesity pathophysiology represent a rational treatment strategy [11].

The clinical success of liraglutide, semaglutide, and more recently tirzepatide strongly supports this concept. Multiple studies have demonstrated meaningful weight loss in post-bariatric patients treated with incretin-based therapies, including those with insufficient weight loss and those with established post-operative recurrence [11-16]. These findings suggest that restoration of appetite control and satiety signaling may allow many patients to recover a substantial proportion of the metabolic benefits initially achieved through surgery.

Proposed Treatment Selection Framework

Management decisions should be individualized according to anatomical findings, severity of recurrence, response to previous interventions, obesity-related disorders, and patient-specific factors (Table 3).

**Table 3:** Proposed selection criteria for incretin-based pharmacotherapy versus revisional bariatric surgery Evidence-informed framework for treatment selection in patients with post-operative weight recurrence following MBS. Incretin-based pharmacotherapy is generally favored when no correctable structural abnormality is identified, whereas endoscopic or revisional surgical intervention is preferred when anatomical failure is demonstrable. Recommendations are based on currently available evidence and expert consensus and should be individualized according to patient characteristics, comorbidity burden, and response to previous interventions. TORe: Transoral outlet reduction; MBS: Metabolic and bariatric surgery

Clinical Scenario	Preferred Strategy
No identifiable anatomical abnormality [4,11]	Incretin-based pharmacotherapy
Mild-to-moderate post-operative weight recurrence [11-15]	Incretin-based pharmacotherapy
Early recurrence (<2 years) without anatomical defect [4,11]	Pharmacotherapy combined with lifestyle intervention
Enlarged gastric pouch [20,21]	Revisional surgery or endoscopic intervention
Dilated gastrojejunal anastomosis [20,21]	TORe or revisional procedure
Gastro-gastric fistula [20,21]	Revisional surgery
Failure of optimized pharmacotherapy [4,21]	Re-evaluation for endoscopic or revisional intervention
Severe anatomical failure [21]	Revisional surgery

Reconsidering the Treatment Algorithm

The availability of highly effective OMMs has prompted reassessment of traditional treatment algorithms. Rather than progressing directly from lifestyle intervention to revisional surgery, an intermediate pharmacologic step may now be appropriate for many patients [4,8].

A contemporary management strategy should begin with comprehensive multidisciplinary evaluation to identify behavioral, psychological, endocrine, metabolic, and anatomical contributors to recurrence [2,4]. Patients with correctable anatomical abnormalities should be considered for endoscopic or surgical revision when appropriate [20,21]. Those without significant structural defects may be ideal candidates for incretin-based pharmacotherapy as a first-line escalation strategy [11-15].

Such an approach aligns with the modern concept of obesity as a chronic disease requiring long-term medical management rather than episodic procedural intervention [3,4]. This strategy does not diminish the importance of revisional surgery but instead promotes its more selective application in patients most likely to derive meaningful and durable benefit.

Potential Advantages of a Pharmacotherapy-First Strategy

A pharmacotherapy-first strategy in appropriately selected patients offers several advantages. Medical therapy avoids the perioperative risks associated with revisional procedures, which carry higher morbidity than primary bariatric operations [21]. It may produce substantial additional weight loss without exposing patients to further anatomical alteration or procedural complications [11-23]. In some patients, a sustained pharmacologic response may delay or ultimately eliminate the need for reoperation.

Furthermore, pharmacotherapy may function as a neoadjuvant strategy. Weight reduction achieved through incretin-based treatment before revisional surgery may improve operative conditions, lower peri-operative risk, and enhance patient selection when surgery is ultimately pursued.

Current Evidence Gaps

Several important uncertainties remain. Most studies evaluating incretin-based therapies after bariatric surgery are observational and involve relatively short follow-up periods [11,12]. The long-term durability of weight loss after treatment discontinuation remains poorly characterized, and direct comparisons between pharmacotherapy and revisional surgery are largely absent from the literature [4]. The optimal timing of treatment initiation also remains undefined; future research should determine whether earlier pharmacologic intervention may better preserve long-term surgical outcomes.

Clinical Perspective

Based on currently available evidence, incretin-based therapies should not be viewed as replacements for revisional bariatric surgery. Rather, they represent valuable additions to the therapeutic armamentarium for managing post-operative recurrence. Their greatest utility is likely in patients without major anatomical abnormalities, where neurohormonal and behavioral mechanisms predominate. As pharmacologic options continue to improve, the threshold for proceeding to revisional surgery will likely evolve accordingly.

The most important contribution of modern incretin-based therapies may ultimately not be the elimination of reoperation, but rather the creation of a therapeutic bridge capable of restoring weight loss, improving metabolic health, and reducing unnecessary procedural exposure in carefully selected patients.

Proposed clinical algorithm for the management of weight recurrence after bariatric surgery

**Figure 2:**
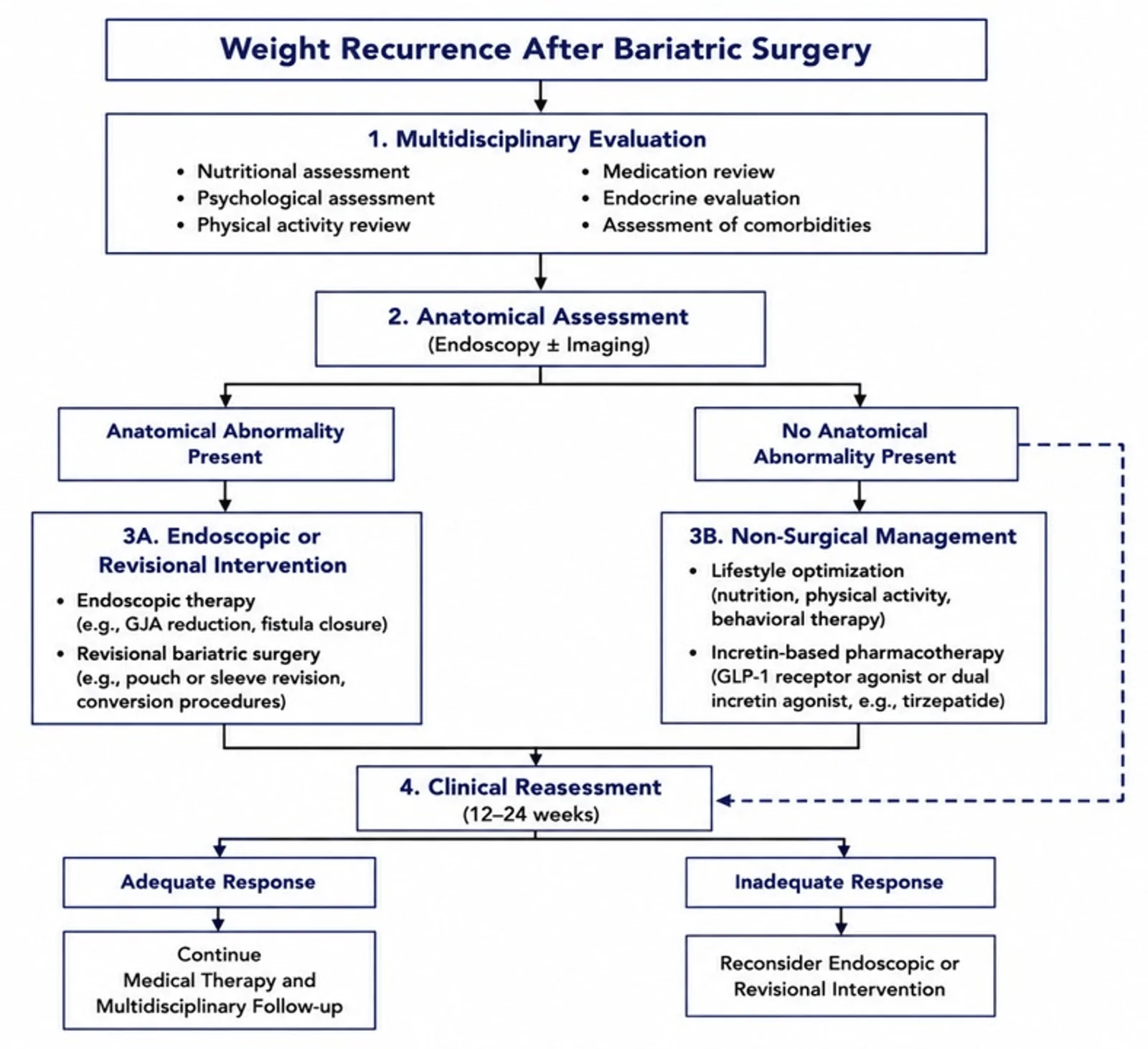
Proposed clinical algorithm for the management of post-operative weight recurrence following MBS Patients presenting with post-operative weight recurrence should undergo comprehensive multidisciplinary evaluation, including nutritional, behavioral, psychological, endocrine, and anatomical assessment. Individuals with demonstrable structural abnormalities may benefit from endoscopic revision or revisional bariatric surgery, whereas those without correctable anatomical defects are candidates for evidence-based OMMs, particularly incretin-based therapies. Ongoing reassessment of treatment response is essential to guide treatment continuation, escalation, or referral for procedural intervention within a personalized management framework. MBS: Metabolic and bariatric surgery; OMM: Obesity management medication

A proposed step-wise management algorithm for post-operative weight recurrence following MBS is presented in Figure 2. Because the mechanisms underlying recurrence are heterogeneous, treatment selection should be guided by identification of the dominant contributing factors rather than by the magnitude of weight recurrence alone [2,4,5].

The proposed algorithm begins with confirmation of clinically significant post-operative recurrence and comprehensive multidisciplinary evaluation. Initial assessment should include nutritional review, behavioral and psychological evaluation, medication reconciliation, and screening for endocrine disorders that may contribute to restoration of body weight [2,4-6].

When clinically indicated, anatomical assessment should be performed using upper gastrointestinal contrast studies, upper endoscopy, or cross-sectional imaging [20,21]. Patients with demonstrable anatomical abnormalities - including gastric pouch enlargement, gastrojejunal anastomotic dilation, gastro-gastric fistula formation, or substantial sleeve dilation - should be considered for endoscopic revision or revisional bariatric surgery as appropriate [20,21].

Patients without correctable structural abnormalities are appropriate candidates for evidence-based OMMs. Current evidence supports GLP-1 RAs and dual incretin agonists as the most effective pharmacologic options currently available for this population [11-23]. Pharmacotherapy should be implemented alongside optimization of dietary habits, physical activity, and behavioral support.

Clinical response should be reassessed periodically. Patients who achieve satisfactory weight reduction and improvement of obesity-related disorders may continue medical therapy with long-term multidisciplinary follow-up. Those who fail to demonstrate adequate clinical improvement despite optimized treatment should undergo re-evaluation and consideration of endoscopic or revisional surgical intervention [4].

This algorithm emphasizes individualized, mechanism-based management and recognizes pharmacotherapy and revisional surgery as complementary rather than competing treatment strategies. The objective is not to replace revisional surgery, but rather to optimize patient selection and ensure that procedural intervention is reserved for those most likely to derive meaningful and durable benefit.

Future directions

The management of post-operative weight recurrence is entering a period of rapid transformation driven by an unprecedented expansion of the obesity pharmacotherapy landscape. As of 2026, more than 100 OMMs are in active clinical development in the United States alone, encompassing a broad range of mechanisms of action, molecular targets, delivery platforms, and dosing schedules [27]. This proliferation of therapeutic options represents both a major opportunity and a source of important unanswered questions for the post-bariatric population.

Standardization of Outcomes and Prospective Evidence

Future research should prioritize the development of standardized, International Federation for the Surgery of Obesity and Metabolic Disorders (IFSO)-aligned definitions for weight recurrence and treatment outcomes in post-bariatric populations. Uniform adoption of patient-centered terminology - including "suboptimal clinical response," "weight recurrence," and "obesity-related disorders" - will facilitate study comparisons, improve meta-analytic precision, and ensure that research framing aligns with contemporary disease models [19].

Prospective randomized controlled trials comparing OMMs with endoscopic and revisional surgical approaches are particularly needed to define the optimal sequencing of interventions, treatment duration, and long-term durability of pharmacologic responses in this population [4]. Such studies will be essential for establishing evidence-based treatment algorithms and identifying patients most likely to benefit from medical versus procedural strategies.

Mechanistic Diversity of the Emerging Pipeline

The agents currently in advanced development extend considerably beyond the GLP-1/GIP dual agonism represented by tirzepatide and encompass multiple mechanistic classes with distinct therapeutic profiles.

Triple-hormone RAs (GLP-1, GIP, and glucagon) represent the most advanced next-generation class. Retatrutide, a once-weekly injectable triple agonist, demonstrated substantial weight reduction in a phase 2 trial, achieving up to 24.2% body weight reduction at 48 weeks [24]. These findings suggest that retatrutide may warrant future evaluation in post-bariatric populations experiencing clinically significant weight recurrence.

GLP-1/glucagon dual agonists constitute a mechanistically distinct class in which glucagon receptor co-agonism enhances energy expenditure and lipid metabolism beyond what is achieved through GLP-1 activation alone. Survodutide (BI 456906) is the most clinically advanced agent in this category; in a phase 2 dose-finding trial published in *The Lancet Diabetes & Endocrinology*, survodutide at 4.8 mg once weekly produced mean weight reduction of 14.9% at 46 weeks, with up to 40% of participants at the highest doses achieving at least 20% weight loss [25]. Further investigation is required to determine the potential role of this therapeutic class in patients with prior MBS.

Oral non-peptide GLP-1 RAs represent another important innovation. Orforglipron, a once-daily oral GLP-1 RA, demonstrated substantial weight reduction in phase 3 clinical trials without the administration restrictions required by oral semaglutide [26]. Oral formulations may improve treatment accessibility and adherence, particularly among patients unwilling or unable to use injectable therapies.

Combination peptide regimens such as CagriSema (cagrilintide plus semaglutide) have also entered late-stage development and represent a complementary strategy targeting satiety through both incretin and non-incretin pathways [27].

Procedure-Specific Pharmacodynamics: An Unresolved Question

One of the most clinically relevant unanswered questions concerns whether the type of prior MBS modifies the response to OMMs. Patients who have undergone RYGB exhibit markedly different post-prandial hormonal profiles than those who have undergone SG, including higher endogenous GLP-1 and PYY responses, altered bile acid metabolism, and substantial changes in gut-brain signaling pathways [9,10].

These physiological differences may influence the magnitude of benefit derived from exogenous incretin therapy, affect gastrointestinal tolerability, or alter pharmacodynamic responses. However, current evidence remains insufficient to determine whether specific agents should be preferentially selected or dosed according to prior bariatric procedure [28].

Future studies specifically designed to evaluate procedure-specific outcomes with individual OMMs would substantially advance clinical decision-making and help establish personalized treatment strategies for this heterogeneous patient population.

Tolerability of OMMs in the Post-bariatric Population

Gastrointestinal adverse effects - including nausea, vomiting, abdominal discomfort, and early satiety - remain the most frequently reported side effects of GLP-1 RAs and dual incretin agonists in both obesity and diabetes populations [17,22]. Available studies in post-bariatric patients have not identified major safety concerns beyond those already recognized in non-surgical populations [11,29].

Whether incretin-based therapies exhibit a distinct tolerability profile in patients with prior MBS remains uncertain. Alterations in gastrointestinal anatomy, nutrient transit, and endogenous gut hormone signaling could theoretically influence treatment tolerability, pharmacokinetics, or dose escalation patterns; however, current evidence is insufficient to draw definitive conclusions [28,29].

Prospective studies specifically designed to evaluate adverse-event profiles, treatment adherence, discontinuation rates, and long-term safety in post-bariatric populations are needed to better define the tolerability of these agents in routine clinical practice.

Toward a Precision Medicine Framework

Advances in precision medicine may ultimately enable more individualized selection among the growing array of available OMMs based on post-operative hormonal phenotype, anatomical configuration, behavioral characteristics, and genetic factors influencing pharmacologic response [19,27].

Rather than applying a uniform escalation strategy, future care models may tailor agent selection to the dominant mechanism driving recurrence in a given patient. Such an approach could allow clinicians to integrate anatomical findings, metabolic status, treatment goals, and patient preferences into a personalized management strategy.

The overarching message conveyed by emerging evidence is that OMMs and MBS are complementary rather than competing therapies. The expanding pharmacologic pipeline does not diminish the indispensable role of surgery in the management of severe obesity and its related disorders; rather, it enriches the therapeutic continuum within which surgical and pharmacologic interventions can be rationally combined and sequenced to optimize long-term outcomes.

## Conclusions

Weight recurrence following MBS is a common and clinically meaningful challenge that reflects the chronic, relapsing biology of obesity, arising from complex interactions among behavioral, psychological, metabolic, neurohormonal, and anatomical factors. Consistent adoption of IFSO-recommended nomenclature - including "suboptimal clinical response," "weight recurrence," and "obesity-related disorders" - is essential to ensure that clinical communication and research framing accurately reflect a disease-centered, non-stigmatizing model of care. The development of incretin-based therapies has substantially expanded the therapeutic landscape for these patients: accumulating evidence supports the efficacy of GLP-1 RAs and dual incretin agonists in producing clinically meaningful weight loss after MBS, with the greatest benefit observed in patients without correctable anatomical abnormalities. Although revisional bariatric surgery remains essential for patients with structural failure of the primary procedure, modern OMMs provide a valuable intermediate strategy capable of restoring weight loss while avoiding the risks associated with reoperation, and these approaches should be viewed as complementary components of a comprehensive treatment algorithm rather than competing therapeutic options.

Looking ahead, the rapid expansion of the obesity pharmacotherapy pipeline - including triple-hormone RAs, GLP-1/glucagon dual agonists, oral non-peptide GLP-1 RAs, and other investigational agents - has the potential to further broaden therapeutic possibilities for the post-bariatric population, though important questions regarding procedure-specific pharmacodynamics, optimal agent selection, treatment sequencing, and long-term durability remain unanswered and warrant dedicated prospective investigation. Based on currently available evidence, modern OMMs have the potential to reduce, defer, or better stratify the need for revisional bariatric surgery in appropriately evaluated patients; their most meaningful contribution may ultimately lie not in replacing surgery, but in completing the continuum of lifelong obesity management that effective long-term care requires.

## References

[1] Eisenberg D, Shikora SA, Aarts E (2022). 2022 American Society for Metabolic and Bariatric Surgery (ASMBS) and International Federation for the Surgery of Obesity and Metabolic Disorders (IFSO): indications for metabolic and bariatric surgery. Surg Obes Relat Dis.

[2] Mechanick JI, Apovian C, Brethauer S (2019). Clinical practice guidelines for the perioperative nutrition, metabolic, and nonsurgical support of patients undergoing bariatric procedures - 2019 update: cosponsored by American Association of Clinical Endocrinologists/American College of Endocrinology, The Obesity Society, American Society For Metabolic & Bariatric Surgery, Obesity Medicine Association, and American Society of Anesthesiologists. Endocr Pract.

[3] Grunvald E, Shah R, Hernaez R (2022). Aga clinical practice guideline on pharmacological interventions for adults with obesity. Gastroenterology.

[4] Haddad A, Suter M, Greve JW (2024). Therapeutic options for recurrence of weight and obesity related complications after metabolic and bariatric surgery: an IFSO position statement. Obes Surg.

[5] King WC, Hinerman AS, Belle SH, Wahed AS, Courcoulas AP (2018). Comparison of the performance of common measures of weight regain after bariatric surgery for association with clinical outcomes. JAMA.

[6] Noria SF, Shelby RD, Atkins KD, Nguyen NT, Gadde KM (2023). Weight regain after bariatric surgery: scope of the problem, causes, prevention, and treatment. Curr Diab Rep.

[7] El Ansari W, Elhag W (2021). Weight regain and insufficient weight loss after bariatric surgery: definitions, prevalence, mechanisms, predictors, prevention and management strategies, and knowledge gaps-a scoping review. Obes Surg.

[8] Lucas E, Simmons O, Tchang B, Aronne L (2022). Pharmacologic management of weight regain following bariatric surgery. Front Endocrinol (Lausanne).

[9] Hutch CR, Sandoval D (2017). The role of GLP-1 in the metabolic success of bariatric surgery. Endocrinology.

[10] Çalık Başaran N, Dotan I, Dicker D (2025). Post metabolic bariatric surgery weight regain: the importance of GLP-1 levels. Int J Obes (Lond).

[11] Dutta D, Nagendra L, Joshi A, Krishnasamy S, Sharma M, Parajuli N (2024). Glucagon-like peptide-1 receptor agonists in post-bariatric surgery patients: a systematic review and meta-analysis. Obes Surg.

[12] Jensen AB, Renström F, Aczél S, Folie P, Biraima-Steinemann M, Beuschlein F, Bilz S (2023). Efficacy of the glucagon-like peptide-1 receptor agonists liraglutide and semaglutide for the treatment of weight regain after bariatric surgery: a retrospective observational study. Obes Surg.

[13] Murvelashvili N, Xie L, Schellinger JN (2023). Effectiveness of semaglutide versus liraglutide for treating post-metabolic and bariatric surgery weight recurrence. Obesity (Silver Spring).

[14] Jamal M, Alhashemi M, Dsouza C, Al-Hassani S, Qasem W, Almazeedi S, Al-Sabah S (2024). Semaglutide and tirzepatide for the management of weight recurrence after sleeve gastrectomy: a retrospective cohort study. Obes Surg.

[15] Mok J, Adeleke MO, Brown A (2023). Safety and efficacy of liraglutide, 3.0 mg, once daily vs placebo in patients with poor weight loss following metabolic surgery: the BARI-OPTIMISE randomized clinical trial. JAMA Surg.

[16] Miras AD, Pérez-Pevida B, Aldhwayan M (2019). Adjunctive liraglutide treatment in patients with persistent or recurrent type 2 diabetes after metabolic surgery: a randomized, double-blind, placebo-controlled trial. Lancet Diabetes Endocrinol.

[17] Wilding JP, Batterham RL, Calanna S (2021). Once-weekly semaglutide in adults with overweight or obesity. N Engl J Med.

[18] King WC, Belle SH, Hinerman AS, Mitchell JE, Steffen KJ, Courcoulas AP (2020). Patient behaviors and characteristics related to weight regain after Roux-en-Y gastric bypass: a multicenter prospective cohort study. Ann Surg.

[19] Salminen P, Kow L, Aminian A (2024). IFSO consensus on definitions and clinical practice guidelines for obesity management - an international Delphi study. Obes Surg.

[20] Jirapinyo P, Kumar N, AlSamman MA, Thompson CC (2020). Five-year outcomes of transoral outlet reduction for the treatment of weight regain after Roux-en-Y gastric bypass. Gastrointest Endosc.

[21] Guarderas X, Cadena-Semanate R, Herrera G, Guerron AD (2020). Surgical approach of weight regain after bariatric surgery. Dig Med Res.

[22] Jastreboff AM, Aronne LJ, Ahmad NN (2022). Tirzepatide once weekly for the treatment of obesity. N Engl J Med.

[23] Aronne LJ, Horn DB, le Roux CW (2025). Tirzepatide as compared with semaglutide for the treatment of obesity. N Engl J Med.

[24] Jastreboff AM, Kaplan LM, Frías JP (2023). Triple-hormone-receptor agonist Retatrutide for obesity - a phase 2 trial. N Engl J Med.

[25] le Roux CW, Steen O, Lucas KJ, Startseva E, Unseld A, Hennige AM (2024). Glucagon and GLP-1 receptor dual agonist survodutide for obesity: a randomised, double-blind, placebo-controlled, dose-finding phase 2 trial. Lancet Diabetes Endocrinol.

[26] Wharton S, Aronne LJ, Stefanski A (2025). Orforglipron, an oral small-molecule GLP-1 receptor agonist for obesity treatment. N Engl J Med.

[27] Müller TD, Blüher M, Tschöp MH, DiMarchi RD (2022). Anti-obesity drug discovery: advances and challenges. Nat Rev Drug Discov.

[28] Wharton S, Kamran E, Muqeem M, Khan A, Christensen RA (2019). The effectiveness and safety of pharmaceuticals to manage excess weight post-bariatric surgery: a systematic literature review. J Drug Assess.

[29] Golzarand M, Toolabi K, Mirmiran P (2025). Obesity medications in patients with recurrent weight gain or suboptimal clinical response following bariatric surgery: a meta-analysis. Int J Obes (Lond).

